# How can clinical pharmacy profession be used at the emergency wards?

**DOI:** 10.1186/1757-7241-20-S2-P25

**Published:** 2012-04-16

**Authors:** Susie Vand, Inge Hermansen

**Affiliations:** 1 Hospital Pharmacy, Aarhus, Denmark

## Background

The aim was to reduce medication errors at the hospital and thereby increase patients’ safety.

Hospital admission and discharge involve a high risk of medication errors, caused by unclear division of responsibilities, lack of communication, frequent exchange of synonyms and analogues, drugs, poly-pharmacy, compliance issues as well as insufficient education and training of the staff.

The pharmacist writes the interventions in the electronic patient record after admission to the medical ward.

## Methods

During 3 month all interventions of the pharmacist were registered. 3 days after making the remarks the pharmacist registered the physician following up. The remarks were arranged in 13 categories for example doses, duplicate prescription, adverse effects, interaction etc. The interventions were registered as accepted, not accepted or no comment.

## Results

The pharmacist checked the journals of 349 patients. Interventions were made on 188 patients corresponding to 54%. The pharmacist wrote 315 interventions for the 188 patients.

The majority of the interventions were dealt with dose (18%), additional treatment suggested (17%), technical problems using the electronic patient record (14%) and inadequate prescription (11%).

Physicians agreed to 51% of the interventions. 10% of the interventions were accepted and modified by a pharmacist. 4% was accepted but has not resulted in an action. This means that there was action on 65% of all interventions.

The follow up remarks regarding duration of treatment and duplicate prescriptions were about 70%. The follow up of inadequate prescription was 63% while the follow up for suggested additional treatment and remarks regarding the electronic patients’ records were 58%.

## Conclusion

Written remarks from pharmacists can be used in the assessment of medication of patients in order to increase the general quality. It can be used as an assisting in optimizing medication treatment for admitted patients.

65% of all interventions are followed up by a physician means a reduction of any possible drug-related problems.

